# Propyl 2-(5-chloro-3-methyl­sulfinyl-1-benzofuran-2-yl)acetate

**DOI:** 10.1107/S1600536809004735

**Published:** 2009-02-13

**Authors:** Hong Dae Choi, Pil Ja Seo, Byeng Wha Son, Uk Lee

**Affiliations:** a Department of Chemistry, Dongeui University, San 24 Kaya-dong Busanjin-gu, Busan 614-714, Republic of Korea; b Department of Chemistry, Pukyong National University, 599-1 Daeyeon 3-dong Nam-gu, Busan 608-737, Republic of Korea

## Abstract

In the title compound, C_14_H_15_ClO_4_S, the O atom and the methyl group of the methyl­sulfinyl substituent lie on opposite sides of the plane of the benzofuran fragment. The crystal structure is stabilized by aromatic π–π inter­actions between the benzene rings of neighbouring mol­ecules [centroid-to-centroid distance = 3.635 (3) Å], and by C—H⋯π inter­actions between a propyl methyl­ene H atom and the furan ring of an adjacent mol­ecule. In addition, the crystal structure exhibits weak inter­molecular C—H⋯O hydrogen bonds.

## Related literature

For the crystal structures of similar alkyl 2-(5-chloro-3-methyl­sulfinyl-1-benzofuran-2-yl)acetates, see: Choi *et al.* (2007 ▶, 2008 ▶).
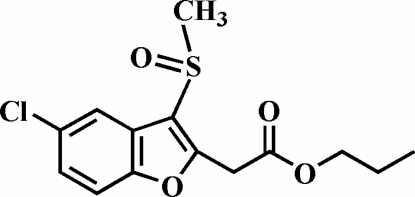


## Experimental

### 

#### Crystal data

C_14_H_15_ClO_4_S
*M* *_r_* = 314.77
Triclinic, 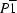

*a* = 8.528 (2) Å
*b* = 9.585 (3) Å
*c* = 10.195 (3) Å
α = 73.452 (4)°
β = 81.773 (5)°
γ = 65.747 (4)°
*V* = 728.0 (4) Å^3^
*Z* = 2
Mo *K*α radiation
μ = 0.42 mm^−1^
*T* = 173 K
0.40 × 0.40 × 0.10 mm

#### Data collection

Bruker SMART CCD diffractometer
Absorption correction: multi-scan (*SADABS*; Sheldrick, 1999 ▶) *T*_min_ = 0.842, *T*_max_ = 0.961
5062 measured reflections
2468 independent reflections
2304 reflections with *I* > 2σ(*I*)
*R*_int_ = 0.029

#### Refinement

*R*[*F* ^2^ > 2σ(*F* ^2^)] = 0.037
*wR*(*F* ^2^) = 0.094
*S* = 1.07
2468 reflections
182 parameters
H-atom parameters constrained
Δρ_max_ = 0.28 e Å^−3^
Δρ_min_ = −0.32 e Å^−3^

### 

Data collection: *SMART* (Bruker, 2001 ▶); cell refinement: *SAINT* (Bruker, 2001 ▶); data reduction: *SAINT*; program(s) used to solve structure: *SHELXS97* (Sheldrick, 2008 ▶); program(s) used to refine structure: *SHELXL97* (Sheldrick, 2008 ▶); molecular graphics: *ORTEP-3* (Farrugia, 1997 ▶) and *DIAMOND* (Brandenburg, 1998 ▶); software used to prepare material for publication: *SHELXL97*.

## Supplementary Material

Crystal structure: contains datablocks global, I. DOI: 10.1107/S1600536809004735/gk2190sup1.cif

Structure factors: contains datablocks I. DOI: 10.1107/S1600536809004735/gk2190Isup2.hkl

Additional supplementary materials:  crystallographic information; 3D view; checkCIF report

## Figures and Tables

**Table 1:** Hydrogen-bond geometry (Å, °)

*D*—H⋯*A*	*D*—H	H⋯*A*	*D*⋯*A*	*D*—H⋯*A*
C12—H12*A*⋯*Cg*2^i^	0.99	2.74	3.666 (3)	155
C3—H3⋯O4^ii^	0.95	2.44	3.353 (2)	160
C5—H5⋯O3^iii^	0.95	2.50	3.373 (2)	152
C9—H9*A*⋯O4^iv^	0.99	2.35	3.321 (2)	166
C9—H9*B*⋯O1^v^	0.99	2.54	3.489 (2)	161

Symmetry codes: (i) 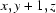; (ii) 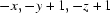; (iii) 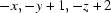; (iv) 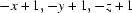; (v) 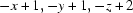. *Cg*2 is the centroid of the C1/C2/C7/O1/C8 furan ring

**Fig. 1.**
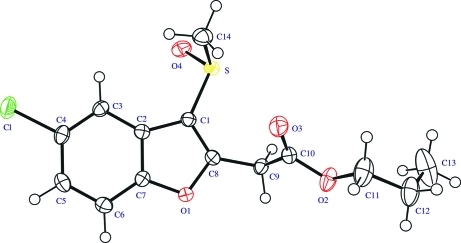
The molecular structure of the title compound, showing displacement ellipsoids drawn at the 50% probability level.

**Fig. 2.**
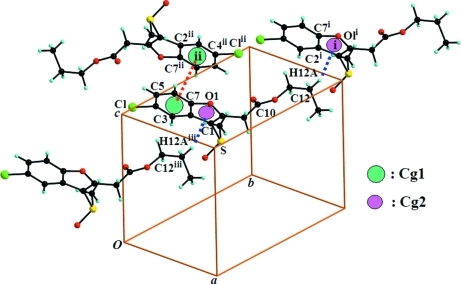
π—π and C—H···π interactions (dotted lines) in the title compound. Cg denotes the ring centroid. [Symmetry code: (i) x, y+1, z; (ii) -x, -y+1, -z+2; (iii) x, y-1, z.]

**Fig. 3.**
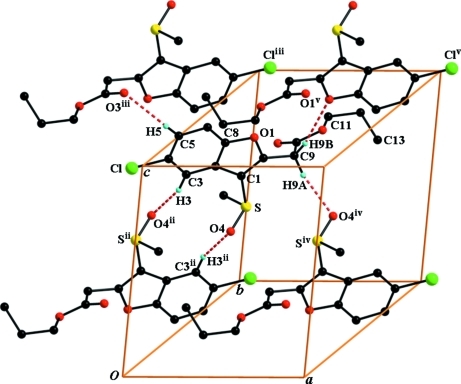
Intermolecular C—H···O hydrogen bonds (dotted lines) in the title compound. [Symmetry code: (ii) -x, -y+1, -z+1; (iii) -x, -y+1, -z+2; (iv) -x+1, -y+1, -z+1; (v) -x+1, -y+1, -z+2.]

## supplementary crystallographic information

### Comment

As a part of our ongoing research on the synthesis and structure of alkyl 2-(5-chloro-3-methylsulfinyl-1-benzofuran-2-yl)acetate analogues, we have described the crystal structures of ethyl 2-(5-chloro-3-methylsulfinyl-1-benzofuran-2-yl)acetate (Choi *et al.*, 2007) and methyl 2-(5-chloro-3-methylsulfinyl-1-benzofuran-2-yl)acetate (Choi *et al.*, 2008). Here we report the crystal structure of the title compound, propyl 2-(5-chloro-3-methylsulfinyl-1-benzofuran-2-yl)acetate (Fig. 1). Regardless of the lengths of the alkyl substituent arrangement of the molecules in the three crystal structures is very similar. The benzofuran unit is essentially planar, with a mean deviation of 0.013 (1) Å from the least-squares plane defined by the nine constituent atoms. The crystal packing (Fig. 2) is stabilized by aromatic π—π interactions between the benzene rings of neighbouring molecules. The Cg1···Cg1^ii^ distance is 3.635 (3) Å (Cg1 is the centroid of the C2–C7 benzene ring; symmetry code as in Fig. 2). The molecular packing is further stabilized by C—H···π interactions between the hydrogen of 12-methylene group and the furan ring of the benzofuran unit, with a C12—H12A···Cg2^i^ separation of 2.74 Å (Table 1 and Fig. 2; Cg2 is the centroid of the C1/C2/C7/O1/C8 furan ring; symmetry code as in Fig. 2). Additionally, four different intermolecular C—H···O hydrogen bonds in the structure are observed (Table 1 & Fig. 3).

### Experimental

77% 3-Chloroperoxybenzoic acid (173 mg, 0.77 mmol) was added in small portions to a stirred solution of propyl 2-(5-chloro-3-methylsulfanyl-1-benzofuran-2-yl)acetate (209 mg, 0.7 mmol) in dichloromethane (20 ml) at 273 K. After being stirred for 3 h at room temperature, the mixture was washed with saturated sodium bicarbonate solution and the organic layer was separated, dried over magnesium sulfate, filtered and concentrated in vacuum. The residue was purified by column chromatography (hexane-ethylacetate, 1:2 v/v) to afford the title compound as a colorless solid [yield 80%, m.p. 399–340 K; R_f_ = 0.51 (hexane-ethyl acetate, 1;2 v/v)]. Single crystals suitable for X-ray diffraction were prepared by evaporation of a solution of the title compound in benzene at room temperature. Spectroscopic analysis: ^1^H NMR (CDCl_3_, 400 MHz) δ 0.92 (t, J = 7.30 Hz, 3H), 1.65-1.74 (m, 2H), 3.05 (s, 3H), 4.03 (s, 2H), 4.13 (t, J = 6.92 Hz, 2H), 7.41 (d, J = 8.72 Hz, 1H), 7.50 (dd, J = 8.72 Hz and J = 1.83 Hz, 1H), 8.03 (d, J = 1.82 Hz, 1H); EI-MS 316 [M+2], 314 [M^+^].

### Refinement

All H atoms were geometrically positioned and refined using a riding model, with C—H = 0.95 Å for the aryl, 0.99 Å for the methylene, and 0.98 Å for the methyl H atoms. U_iso_(H) = 1.2U_eq_(C) for the aryl and methylene H atoms, and 1.5U_eq_(C) for the methyl H atoms.

### Crystal data

**Table:**

C_14_H_15_ClO_4_S	*Z* = 2
*M**_r_* = 314.77	*F*(000) = 328
Triclinic, *P*1	*D*_x_ = 1.436 Mg m^−^^3^
Hall symbol: -P 1	Mo *K*α radiation, λ = 0.71073 Å
*a* = 8.528 (2) Å	Cell parameters from 4784 reflections
*b* = 9.585 (3) Å	θ = 2.4–28.3°
*c* = 10.195 (3) Å	µ = 0.42 mm^−^^1^
α = 73.452 (4)°	*T* = 173 K
β = 81.773 (5)°	Block, colorless
γ = 65.747 (4)°	0.40 × 0.40 × 0.10 mm
*V* = 728.0 (4) Å^3^	

### Data collection

**Table:**

Bruker SMART CCD diffractometer	2468 independent reflections
Radiation source: fine-focus sealed tube	2304 reflections with *I* > 2σ(*I*)
graphite	*R*_int_ = 0.029
Detector resolution: 10.0 pixels mm^-1^	θ_max_ = 25.0°, θ_min_ = 2.1°
φ and ω scans	*h* = −10→10
Absorption correction: multi-scan (*SADABS*; Sheldrick, 1999)	*k* = −11→11
*T*_min_ = 0.842, *T*_max_ = 0.961	*l* = −12→12
5062 measured reflections	

### Refinement

**Table:**

Refinement on *F*^2^	Primary atom site location: structure-invariant direct methods
Least-squares matrix: full	Secondary atom site location: difference Fourier map
*R*[*F*^2^ > 2σ(*F*^2^)] = 0.037	Hydrogen site location: inferred from neighbouring sites
*wR*(*F*^2^) = 0.094	H-atom parameters constrained
*S* = 1.07	*w* = 1/[σ^2^(*F*_o_^2^) + (0.0381*P*)^2^ + 0.4505*P*] where *P* = (*F*_o_^2^ + 2*F*_c_^2^)/3
2468 reflections	(Δ/σ)_max_ < 0.001
182 parameters	Δρ_max_ = 0.28 e Å^−^^3^
0 restraints	Δρ_min_ = −0.32 e Å^−^^3^

### Special details

**Table:**

Geometry. All esds (except the esd in the dihedral angle between two l.s. planes) are estimated using the full covariance matrix. The cell esds are taken into account individually in the estimation of esds in distances, angles and torsion angles; correlations between esds in cell parameters are only used when they are defined by crystal symmetry. An approximate (isotropic) treatment of cell esds is used for estimating esds involving l.s. planes.
Refinement. Refinement of F^2^ against ALL reflections. The weighted R-factor wR and goodness of fit S are based on F^2^, conventional R-factors R are based on F, with F set to zero for negative F^2^. The threshold expression of F^2^ > 2sigma(F^2^) is used only for calculating R-factors(gt) etc. and is not relevant to the choice of reflections for refinement. R-factors based on F^2^ are statistically about twice as large as those based on F, and R- factors based on ALL data will be even larger.

### Fractional atomic coordinates and isotropic or equivalent isotropic displacement parameters (Å^2^)

**Table:**

	*x*	*y*	*z*	*U*_iso_*/*U*_eq_	
S	0.23799 (6)	0.59458 (6)	0.53751 (4)	0.02410 (15)	
Cl	−0.18859 (7)	0.22051 (7)	0.88822 (6)	0.03715 (17)	
O1	0.33263 (16)	0.45103 (15)	0.93160 (12)	0.0223 (3)	
O2	0.46225 (18)	0.85866 (17)	0.78142 (17)	0.0349 (4)	
O3	0.21787 (18)	0.86811 (17)	0.71372 (15)	0.0338 (4)	
O4	0.24182 (19)	0.46691 (18)	0.47748 (14)	0.0321 (4)	
C1	0.2395 (2)	0.5204 (2)	0.71715 (18)	0.0206 (4)	
C2	0.1483 (2)	0.4276 (2)	0.80462 (18)	0.0203 (4)	
C3	0.0205 (2)	0.3780 (2)	0.78783 (19)	0.0228 (4)	
H3	−0.0289	0.4059	0.7016	0.027*	
C4	−0.0300 (2)	0.2866 (2)	0.9031 (2)	0.0249 (4)	
C5	0.0395 (3)	0.2434 (2)	1.0326 (2)	0.0266 (4)	
H5	0.0019	0.1786	1.1082	0.032*	
C6	0.1625 (2)	0.2954 (2)	1.04984 (19)	0.0252 (4)	
H6	0.2100	0.2693	1.1366	0.030*	
C7	0.2134 (2)	0.3870 (2)	0.93509 (19)	0.0209 (4)	
C8	0.3462 (2)	0.5306 (2)	0.79717 (18)	0.0211 (4)	
C9	0.4628 (2)	0.6175 (2)	0.76992 (19)	0.0229 (4)	
H9A	0.5337	0.5986	0.6860	0.028*	
H9B	0.5416	0.5757	0.8470	0.028*	
C10	0.3636 (2)	0.7945 (2)	0.75211 (19)	0.0244 (4)	
C11	0.3854 (3)	1.0300 (3)	0.7669 (3)	0.0439 (6)	
H11A	0.3053	1.0552	0.8453	0.053*	
H11B	0.3204	1.0848	0.6815	0.053*	
C12	0.5296 (3)	1.0819 (3)	0.7631 (3)	0.0451 (6)	
H12A	0.4800	1.1959	0.7603	0.054*	
H12B	0.5932	1.0244	0.8489	0.054*	
C13	0.6534 (5)	1.0561 (4)	0.6453 (4)	0.0753 (10)	
H13A	0.5929	1.1158	0.5596	0.090*	
H13B	0.7052	0.9432	0.6478	0.090*	
H13C	0.7439	1.0921	0.6509	0.090*	
C14	0.0216 (3)	0.7411 (2)	0.5235 (2)	0.0318 (5)	
H14A	0.0002	0.8006	0.4277	0.048*	
H14B	0.0062	0.8137	0.5798	0.048*	
H14C	−0.0595	0.6891	0.5554	0.048*	

### Atomic displacement parameters (Å^2^)

**Table:**

	*U*^11^	*U*^22^	*U*^33^	*U*^12^	*U*^13^	*U*^23^
S	0.0256 (3)	0.0286 (3)	0.0184 (2)	−0.0127 (2)	−0.00144 (18)	−0.00292 (19)
Cl	0.0348 (3)	0.0380 (3)	0.0473 (3)	−0.0243 (2)	−0.0025 (2)	−0.0075 (2)
O1	0.0237 (7)	0.0252 (7)	0.0198 (6)	−0.0117 (6)	−0.0034 (5)	−0.0035 (5)
O2	0.0254 (8)	0.0228 (7)	0.0590 (10)	−0.0088 (6)	−0.0060 (7)	−0.0128 (7)
O3	0.0249 (8)	0.0292 (8)	0.0437 (9)	−0.0098 (6)	−0.0075 (6)	−0.0019 (7)
O4	0.0341 (8)	0.0393 (8)	0.0260 (7)	−0.0132 (7)	−0.0016 (6)	−0.0143 (6)
C1	0.0228 (9)	0.0198 (9)	0.0190 (9)	−0.0080 (7)	−0.0015 (7)	−0.0047 (7)
C2	0.0211 (9)	0.0173 (9)	0.0207 (9)	−0.0054 (7)	−0.0012 (7)	−0.0053 (7)
C3	0.0234 (10)	0.0217 (9)	0.0254 (10)	−0.0086 (8)	−0.0026 (7)	−0.0085 (8)
C4	0.0214 (10)	0.0210 (10)	0.0346 (11)	−0.0088 (8)	0.0007 (8)	−0.0105 (8)
C5	0.0268 (10)	0.0209 (10)	0.0274 (10)	−0.0088 (8)	0.0031 (8)	−0.0019 (8)
C6	0.0252 (10)	0.0244 (10)	0.0225 (9)	−0.0074 (8)	−0.0020 (8)	−0.0033 (8)
C7	0.0196 (9)	0.0192 (9)	0.0240 (9)	−0.0069 (7)	−0.0013 (7)	−0.0062 (7)
C8	0.0220 (9)	0.0196 (9)	0.0204 (9)	−0.0074 (8)	−0.0004 (7)	−0.0039 (7)
C9	0.0202 (9)	0.0244 (10)	0.0261 (9)	−0.0103 (8)	−0.0015 (7)	−0.0064 (8)
C10	0.0250 (10)	0.0269 (10)	0.0233 (9)	−0.0139 (9)	0.0003 (8)	−0.0039 (8)
C11	0.0322 (12)	0.0236 (11)	0.0760 (18)	−0.0075 (10)	−0.0016 (11)	−0.0177 (11)
C12	0.0357 (13)	0.0242 (11)	0.0760 (18)	−0.0114 (10)	−0.0014 (12)	−0.0139 (11)
C13	0.080 (2)	0.0462 (17)	0.093 (2)	−0.0344 (16)	0.0380 (19)	−0.0128 (16)
C14	0.0314 (11)	0.0270 (11)	0.0325 (11)	−0.0069 (9)	−0.0078 (9)	−0.0043 (9)

### Geometric parameters (Å, °)

**Table:**

S—O4	1.5038 (16)	C6—C7	1.382 (3)
S—C1	1.7656 (18)	C6—H6	0.9500
S—C14	1.797 (2)	C8—C9	1.493 (3)
Cl—C4	1.752 (2)	C9—C10	1.522 (3)
O1—C7	1.379 (2)	C9—H9A	0.9900
O1—C8	1.380 (2)	C9—H9B	0.9900
O2—C10	1.333 (2)	C11—C12	1.496 (3)
O2—C11	1.468 (3)	C11—H11A	0.9900
O3—C10	1.207 (2)	C11—H11B	0.9900
C1—C8	1.354 (3)	C12—C13	1.487 (4)
C1—C2	1.452 (3)	C12—H12A	0.9900
C2—C3	1.403 (3)	C12—H12B	0.9900
C2—C7	1.403 (3)	C13—H13A	0.9800
C3—C4	1.383 (3)	C13—H13B	0.9800
C3—H3	0.9500	C13—H13C	0.9800
C4—C5	1.407 (3)	C14—H14A	0.9800
C5—C6	1.383 (3)	C14—H14B	0.9800
C5—H5	0.9500	C14—H14C	0.9800
			
O4—S—C1	106.94 (9)	C10—C9—H9A	109.2
O4—S—C14	106.77 (10)	C8—C9—H9B	109.2
C1—S—C14	98.32 (9)	C10—C9—H9B	109.2
C7—O1—C8	106.32 (14)	H9A—C9—H9B	107.9
C10—O2—C11	116.86 (16)	O3—C10—O2	124.34 (19)
C8—C1—C2	107.36 (16)	O3—C10—C9	125.35 (19)
C8—C1—S	122.84 (15)	O2—C10—C9	110.29 (16)
C2—C1—S	129.51 (15)	O2—C11—C12	107.24 (18)
C3—C2—C7	119.33 (17)	O2—C11—H11A	110.3
C3—C2—C1	136.18 (17)	C12—C11—H11A	110.3
C7—C2—C1	104.48 (17)	O2—C11—H11B	110.3
C4—C3—C2	116.72 (17)	C12—C11—H11B	110.3
C4—C3—H3	121.6	H11A—C11—H11B	108.5
C2—C3—H3	121.6	C13—C12—C11	114.7 (3)
C3—C4—C5	123.34 (19)	C13—C12—H12A	108.6
C3—C4—Cl	118.78 (15)	C11—C12—H12A	108.6
C5—C4—Cl	117.87 (15)	C13—C12—H12B	108.6
C6—C5—C4	119.91 (18)	C11—C12—H12B	108.6
C6—C5—H5	120.0	H12A—C12—H12B	107.6
C4—C5—H5	120.0	C12—C13—H13A	109.5
C7—C6—C5	117.01 (18)	C12—C13—H13B	109.5
C7—C6—H6	121.5	H13A—C13—H13B	109.5
C5—C6—H6	121.5	C12—C13—H13C	109.5
O1—C7—C6	125.69 (17)	H13A—C13—H13C	109.5
O1—C7—C2	110.66 (16)	H13B—C13—H13C	109.5
C6—C7—C2	123.65 (18)	S—C14—H14A	109.5
C1—C8—O1	111.16 (17)	S—C14—H14B	109.5
C1—C8—C9	132.90 (17)	H14A—C14—H14B	109.5
O1—C8—C9	115.80 (16)	S—C14—H14C	109.5
C8—C9—C10	112.11 (15)	H14A—C14—H14C	109.5
C8—C9—H9A	109.2	H14B—C14—H14C	109.5
			
O4—S—C1—C8	132.24 (16)	C3—C2—C7—O1	−177.81 (15)
C14—S—C1—C8	−117.31 (17)	C1—C2—C7—O1	1.2 (2)
O4—S—C1—C2	−40.91 (19)	C3—C2—C7—C6	2.1 (3)
C14—S—C1—C2	69.54 (19)	C1—C2—C7—C6	−178.94 (17)
C8—C1—C2—C3	178.0 (2)	C2—C1—C8—O1	0.0 (2)
S—C1—C2—C3	−8.0 (3)	S—C1—C8—O1	−174.43 (12)
C8—C1—C2—C7	−0.7 (2)	C2—C1—C8—C9	−175.32 (19)
S—C1—C2—C7	173.25 (14)	S—C1—C8—C9	10.2 (3)
C7—C2—C3—C4	−1.9 (3)	C7—O1—C8—C1	0.7 (2)
C1—C2—C3—C4	179.6 (2)	C7—O1—C8—C9	176.90 (15)
C2—C3—C4—C5	0.3 (3)	C1—C8—C9—C10	72.8 (3)
C2—C3—C4—Cl	−179.76 (13)	O1—C8—C9—C10	−102.43 (18)
C3—C4—C5—C6	1.2 (3)	C11—O2—C10—O3	0.8 (3)
Cl—C4—C5—C6	−178.73 (15)	C11—O2—C10—C9	179.06 (18)
C4—C5—C6—C7	−1.1 (3)	C8—C9—C10—O3	−26.0 (3)
C8—O1—C7—C6	178.95 (18)	C8—C9—C10—O2	155.70 (16)
C8—O1—C7—C2	−1.16 (19)	C10—O2—C11—C12	−163.3 (2)
C5—C6—C7—O1	179.33 (17)	O2—C11—C12—C13	62.5 (3)
C5—C6—C7—C2	−0.6 (3)		

### Hydrogen-bond geometry (Å, °)

**Table:**

*D*—H···*A*	*D*—H	H···*A*	*D*···*A*	*D*—H···*A*
C12—H12A···Cg2^i^	0.99	2.74	3.666 (3)	155
C3—H3···O4^ii^	0.95	2.44	3.353 (2)	160
C5—H5···O3^iii^	0.95	2.50	3.373 (2)	152
C9—H9A···O4^iv^	0.99	2.35	3.321 (2)	166
C9—H9B···O1^v^	0.99	2.54	3.489 (2)	161

Symmetry codes: (i) *x*, *y*+1, *z*; (ii) −*x*, −*y*+1, −*z*+1; (iii) −*x*, −*y*+1, −*z*+2; (iv) −*x*+1, −*y*+1, −*z*+1; (v) −*x*+1, −*y*+1, −*z*+2.
